# Efficacy comparison of clown care therapy versus virtual reality distraction on venipuncture-induced anxiety in preschool children: a retrospective cohort study

**DOI:** 10.3389/fmed.2026.1785095

**Published:** 2026-03-18

**Authors:** Yannan Cheng, Chang-cui Chen, Jingfang Hong

**Affiliations:** 1 School of Nursing, Anhui Medical University, Hefei, China; 2 Department of Pediatrics, Dongcheng Campus, The First Affiliated Hospital of Anhui Medical University, Hefei, China

**Keywords:** anxiety, clown care therapy, preschool children, venous blood sampling, virtual reality distraction

## Abstract

**Background:**

Venous blood sampling is a common medical procedure in preschool children that often induces significant anxiety, affecting procedural success and long-term medical adaptation. Clown care therapy (CCT) and virtual reality distraction (VRD) are non-pharmacological interventions with potential to alleviate children’s anxiety, but existing research primarily focuses on Western populations, lacking direct comparisons in the Chinese cultural context, and often relies on subjective scales with insufficient use of objective physiological indicators.

**Objective:**

To compare the efficacy of CCT and VRD in reducing anxiety during venous blood sampling in preschool children, and evaluate their clinical application value using both subjective and objective metrics.

**Methods:**

This single-center, retrospective cohort study was conducted at Dongcheng District, the First Affiliated Hospital of Anhui Medical University. Medical records of 220 preschool children aged 3–6 years who underwent venous blood sampling between January 2022 and January 2025 were reviewed. Based on the non-pharmacological distraction method administered as part of routine clinical care and documented in their medical records, participants were categorized into two groups: the CCT group (*n* = 110), which received interactive play by medical clowns, and the VRD group (*n* = 110), which used virtual reality devices during the procedure. Outcomes included subjective anxiety scores (modified Yale Preoperative Anxiety Scale, m-YPAS), objective physiological indicators (heart rate, crying duration, cortisol changes), clinical operation indicators (cooperation level, first-attempt success rate, sampling time), and parental satisfaction. Inter-group comparisons were conducted to assess intervention efficacy.

**Results:**

The CCT group suggested significantly lower anxiety scores during blood sampling (*t* = 4.128, *p* < 0.001), smaller increase in heart rate (*t* = 5.217, *p* < 0.001), shorter crying duration (*t* = 6.384, *p* < 0.001), and less cortisol change (*t* = 3.724, *p* < 0.001) compared to the VRD group. Cooperation level was better in the CCT group (*p* < 0.001), first-attempt success rate was higher (χ^2^ = 5.143, *p* = 0.023), and sampling time was shorter (*t* = 4.926, *p* < 0.001). Parental satisfaction was higher in the CCT group (*p* < 0.001). Multivariate analysis indicated CCT was independently associated with greater anxiety reduction (*p* < 0.001).

**Conclusion:**

In this retrospective analysis, CCT was associated with better outcomes than VRD in alleviating anxiety induced by venous blood sampling in preschool children, significantly improving subjective anxiety, physiological stress responses, clinical efficiency, and parental satisfaction. Its interactive, human-centered nature may be particularly acceptable in this setting, though this requires further investigation.

## 1 Introduction

Clown Care Therapy (CCT) is a non-pharmacological intervention that employs professionally trained medical clowns to reduce pediatric medical anxiety through interactive games, music, and humorous behaviors. Its application in perioperative and pain management settings has been supported by multiple studies (1). Virtual Reality Distraction (VRD), on the other hand, utilizes immersive audiovisual experiences to divert children’s attention away from medical procedures. It has been widely adopted in recent years to alleviate procedure-related anxiety in pediatric populations, with suggested efficacy in contexts such as dental treatments and vaccinations (2).

Venous blood sampling represents one of the most common medical stressors among preschool-aged children, frequently eliciting significant anxiety responses manifested as crying, physical resistance, and alterations in physiological parameters. These reactions may compromise procedural efficiency and long-term adaptive healthcare behaviors (3). Current pediatric anxiety management guidelines recommend non-pharmacological interventions as first-line strategies, particularly in young children (4).

Although both CCT and VRD show potential in reducing pediatric anxiety, several limitations persist in the existing literature. Most trials have compared a single active intervention against routine care rather than directly comparing the efficacy of two active modalities, thereby offering limited guidance for selecting optimal interventions in clinical practice (5). Moreover, the majority of studies have been conducted in Western populations, whose cultural contexts and healthcare environments differ substantially from those in China. Given that humor perception and acceptance of virtual reality are strongly influenced by cultural factors, region-specific evidence is urgently needed (6).

Regarding outcome measures, prior research has relied heavily on subjective parent- or nurse-reported scales, which are susceptible to assessment bias. Objective physiological parameters such as heart rate variability (HRV) and salivary cortisol levels have not been systematically integrated into pediatric anxiety research (7). More critically, there is a lack of stratified analyses examining differential intervention responses among children with varying baseline anxiety levels, hindering the development of individualized recommendations (8).

This retrospective cohort study aimed to compare the outcomes associated with CCT and VRD in reducing venipuncture-related anxiety among children aged 3–6 years, based on clinical data collected in a standardized setting at Dongcheng District, the First Affiliated Hospital of Anhui Medical University. A multidimensional anxiety assessment framework is constructed by combining subjective measures (the modified Yale Preoperative Anxiety Scale, m-YPAS) with objective physiological indicators (HRV and salivary cortisol) (9). All interventions are delivered by standardized trained medical clown teams and VR operators to ensure procedural consistency (10). Furthermore, subgroup analyses based on baseline anxiety levels and prior medical experience are conducted to identify differential responder profiles (11).

This study aims to provide high-quality evidence for non-pharmacological anxiety management within the Chinese healthcare context and to explore pathways for optimizing culturally adapted interventional models.

## 2 Materials and methods

### 2.1 General information

Medical records of children aged 3–6 years who underwent elective venous blood sampling between January 2022 and January 2025 were screened. A total of 220 children who met the inclusion and exclusion criteria and for whom complete data for the studied variables were available were included in the final analysis. The allocation to the CCT or VRD group was based on the intervention documented as having been administered during the clinical encounter. The choice of intervention in clinical practice was non-randomized and influenced by a combination of factors including, but not limited to, real-time availability of the medical clown team or VR equipment, staffing schedules, and informal assessment of child receptiveness by the nursing staff. All venipuncture procedures were performed by experienced pediatric nurses following standard protocols.

The sample size of 110 patients per group was considered adequate for retrospective analysis. A *post hoc* power analysis was conducted using G*Power software (Version 3.1.9). With an observed effect size of Cohen’s d = 0.6 (derived from preliminary data comparing m-YPAS scores between distraction modalities), a total sample of 220 participants provided more than 90% statistical power at a two-tailed *α* level of 0.05, accounting for complete data availability in the reviewed records.

### 2.2 Inclusion and exclusion criteria

#### 2.2.1 Inclusion criteria

- Age 3–6 years;
- Scheduled for elective venipuncture (e.g., outpatient or inpatient testing);
- Availability of complete clinical record data for the variables of interest;
- Absence of language or hearing impairments, with basic communication abilities.

#### 2.2.2 Exclusion criteria

- Severe underlying conditions (e.g., congenital heart disease, epilepsy, autism spectrum disorder);
- Recent use (within one week) of sedatives or anxiolytics;
- History of allergy or discomfort toward clown props or virtual reality equipment;
- Parental refusal to participate or inability to comply with follow-up;
- Emergency or urgent venipuncture.

These criteria were established based on routine clinical practice to ensure study safety and feasibility.

### 2.3 Equipment and materials

#### 2.3.1 Clown care therapy equipment

Standard clown attire (colorful clothing, red nose) and interactive props (e.g., balloons, toy instruments) were used. All clown roles were performed by trained medical clowns (typically nurses or volunteers) to ensure age-appropriate interactions. Equipment was simple, low-cost, and sterilized before and after use in compliance with hospital infection control standards.

#### 2.3.2 Virtual reality distraction equipment

Oculus Quest 2 headsets (Meta Platforms, Inc.) were used, preloaded with distraction content suitable for preschoolers (e.g., segments of Peppa Pig or simple interactive games). Content was reviewed and approved by the hospital medical affairs department to avoid excessive visual or auditory stimulation. Devices were maintained and charged according to manufacturer guidelines to ensure stable performance.

#### 2.3.3 Other equipment

Standard venipuncture supplies (e.g., BD Vacutainer needles) and vital sign monitoring devices (Philips IntelliVue MP50 monitors for heart rate measurement) were employed. All equipment was regularly calibrated and maintained per clinical protocols to ensure data accuracy.

### 2.4 Study methods

Study Design and Intervention Assignment: This was a single-center, retrospective cohort study. The interventions (CCT and VRD) were implemented as part of the hospital’s standard nursing care protocol for anxiety management during pediatric venipuncture. The selection of a specific intervention for a given child was not randomized but was determined pragmatically during the clinical encounter. The primary determinants were the scheduled availability of the trained medical clown team (which operated on specific weekdays) and the on-site availability of functional VR equipment. Parental preference expressed at check-in and the nurse’s initial brief assessment of the child’s temperament (e.g., very shy vs. curious about technology) could also influence the final choice. This process reflects real-world clinical decision-making. Due to the obvious nature of the interventions, blinding of the children, parents, or care providers was not possible. Data collectors extracting information from records were not involved in the original clinical care and were unaware of the study hypothesis.

#### 2.4.1 Intervention protocol

##### 2.4.1.1 Observation group

Five minutes prior to venipuncture, a standardized interactive intervention was initiated by a trained medical clown and continued throughout the procedure. The protocol mandated: (1) Initial Engagement: The clown approached the child using a soft voice and friendly gestures, introducing themselves and their purpose. (2) Structured Activities: A sequence of age-appropriate, non-threatening activities was employed, prioritizing the child’s interest. This included: (a) Storytelling: Short (1–2 min), simple stories with positive themes (e.g., adventures, friendly animals). (b) Singing: Familiar, gentle nursery rhymes or children’s songs, often involving simple gestures or encouraging the child to hum along. (c) Simple Magic Tricks: Illusions without sudden noises or frightening elements (e.g., disappearing a small soft ball, producing a colorful scarf). (3) Procedural Support: During needle insertion, the clown used direct, positive distraction (e.g., making eye contact, blowing bubbles together, engaging in a simple call-and-response game) rather than humor potentially misinterpreted as mocking. The clown’s positioning was standardized to be within the child’s line of sight but not obstructing the phlebotomist.

##### 2.4.1.2 Control group

Immediately before positioning for venipuncture (approximately 1 min prior to needle insertion), the VR headset was fitted and activated by a trained operator. The child watched a single, continuous pre-selected video segment. The content was chosen from a predefined library of 3 options, all deemed highly engaging and neutral for this age group based on pilot testing: (1) A 5-min segment from the animated series Peppa Pig (episodes involving park visits or family activities, excluding medical themes). (2) A 5-min video of a gentle aquarium tour with swimming fish and calming music. (3) A 5-min interactive but passive game where the child could watch colorful bubbles float and pop by gazing at them (no controller used). The video volume was set at 60% of maximum device output. The intervention continued until the blood draw was complete and the needle withdrawn. The operator remained present to ensure device stability and safety.

##### 2.4.1.3 Standardization of phlebotomy

All venipuncture procedures were performed by the same team of experienced nurses to minimize operator-dependent variability. The antecubital vein was uniformly selected as the puncture site. A standardized technique was employed, emphasizing first-attempt success and a consistent needle insertion angle.

##### 2.4.1.4 Ethical considerations

This retrospective study was approved by the Hospital Ethics Committee with a waiver of individual informed consent for the analysis of anonymized clinical data, as it involved no more than minimal risk to participants. However, for the original clinical administration of CCT or VRD, verbal assent from the child and verbal permission from the parent/guardian were obtained as per hospital routine care policy.

### 2.5 Observation indicators

1. Anxiety score: Assessed using the Modified Yale Preoperative Anxiety Scale (m-YPAS) (12). This scale comprises four domains (activity, vocalizations, emotional expressivity, and state of alertness), with a total score ranging from 0 to 100; higher scores indicate greater anxiety. Assessments were conducted at baseline (pre-venipuncture), during venipuncture, and 5 min post-venipuncture. Ratings were recorded in real-time by trained observers to ensure consistency.
2. Heart rate variability: Heart rate (beats per minute, bpm) was monitored using a Philips IntelliVue MP50 monitor as an objective physiological correlate of anxiety. Measurements were taken pre-venipuncture, during venipuncture, and 5 min post-venipuncture. Each recording lasted for 1 min, with the average value calculated.
3. Crying duration: An independent observer used a stopwatch to record the duration of crying (in seconds), from needle insertion until the child ceased crying, reflecting behavioral distress.
4. Cooperation level: Evaluated immediately after the procedure by the phlebotomy nurse using a 4-point cooperation scale (1 = fully cooperative, 2 = mildly uncooperative, 3 = moderately uncooperative, 4 = extremely uncooperative). This simple scale is widely utilized in pediatric procedural anxiety research.
5. Parental satisfaction: Assessed post-venipuncture using a 5-point Likert scale questionnaire (1 = very dissatisfied, 2 = dissatisfied, 3 = neutral, 4 = satisfied, 5 = very satisfied) to gauge parental acceptance of the intervention.
6. Phlebotomy-related metrics: Included first-attempt success (defined as successful blood collection on the initial needle insertion, recorded as yes/no) and total procedure time (in seconds, from skin disinfection to completion of blood draw). These metrics directly reflect clinical procedural efficiency.

All data were collected by observers who underwent unified training. Inter-rater reliability was confirmed in a pilot study, achieving a Cohen’s *κ* value greater than 0.8.

### 2.6 Statistical analysis

Data analysis was performed using IBM SPSS Statistics software (Version 26). The normality of continuous data was assessed using the Shapiro–Wilk test. Data conforming to a normal distribution are presented as mean ± standard deviation (SD) and were compared between groups using the independent samples *t*-test. Non-normally distributed data are presented as median (interquartile range, IQR) and were compared using the Mann–Whitney *U* test. Within-group comparisons across time points were analyzed using the paired *t*-test or the Wilcoxon signed-rank test, as appropriate. Changes in anxiety scores over time were analyzed using repeated-measures analysis of variance (ANOVA). Categorical data are described using frequencies and percentages, with group comparisons made using the Chi-square test or Fisher’s exact test. The significance level (*α*) was set at 0.05 for two-tailed tests. For logistic regression, the assumption of linearity for continuous log-odds was checked using the Box-Tidwell procedure, and no violations were detected. Multicollinearity was assessed via variance inflation factors (all VIF < 2).

## 3 Results

### 3.1 Comparison of baseline characteristics between groups

No statistically significant differences were observed in baseline characteristics between the CCT and VRD groups, including age, sex, body weight, previous venipuncture experience, parental education level, baseline heart rate, cortisol level, m-YPAS score, household income, only-child status, primary caregiver, fasting time before blood sampling, daily screen time, allergy history, and oxygen saturation (*t*-values ranged from 1.214 to 1.875; χ^2^ values ranged from 0.291 to 0.514; all *p* > 0.05). These findings indicate that the baseline data were well-balanced and comparable between the two groups (see Table 1).

**Table 1:** Comparison of baseline characteristics between groups.

Variable	CCT Group (*n* = 110)	VRD Group (*n* = 110)	Statistics	*p*-value
Age (years)	4.5 ± 1.2	4.7 ± 1.1	*t* = 1.324	0.187
Sex (male/female)	58/52	62/48	χ^2^ = 0.327	0.567
Body weight (kg)	18.7 ± 3.2	19.2 ± 2.9	*t* = 1.286	0.2
Previous venipuncture experience (yes/no)	67/43	71/39	χ^2^ = 0.382	0.536
Parental education level (high school or below/college or above)	49/61	53/57	χ^2^ = 0.291	0.59
Baseline heart rate (beats/min)	98.6 ± 10.3	101.2 ± 9.8	*t* = 1.875	0.062
Baseline cortisol (nmol/L)	5.8 ± 1.5	6.1 ± 1.6	*t* = 1.482	0.14
Baseline m-YPAS score	38.4 ± 8.7	40.1 ± 9.2	*t* = 1.427	0.155
Baseline oxygen saturation (%)	98.5 ± 1.2	98.3 ± 1.4	*t* = 1.214	0.226

### 3.2 Comparison of anxiety scores between groups at different time points

Prior to the intervention, no significant difference was observed in m-YPAS scores between the two groups (*t* = 1.427, *p* = 0.155). During blood collection, the VRD group exhibited significantly higher scores compared to the CCT group (*t* = 4.128, *p* < 0.001). At 5 min post-blood collection, the scores between groups were comparable (*t* = 1.572, *p* = 0.117). Significant effects were identified for time (*F* = 185.326, *p* < 0.001), group (*F* = 9.417, *p* = 0.002), and interaction (*F* = 6.885, *p* = 0.001), indicating that anxiety scores varied over time and that the intervention effects differed between groups. Details are presented in Table 2.

**Table 2:** Comparison of m-YPAS scores between groups at different time points (Mean ± SD).

Group	Pre-blood collection	During blood collection	5-min post-blood collection	*F*-value	*p*-value
CCT Group	38.4 ± 8.7	52.3 ± 10.2	41.6 ± 9.3	Time: *F* = 185.326	<0.001
VRD Group	40.1 ± 9.2	58.7 ± 11.5	43.2 ± 8.9	Group: *F* = 9.417	0.002
*t*-value	1.427	4.128	1.572	Interaction: *F* = 6.885	0.001
*p*-value	0.155	<0.001	0.117		

### 3.3 Comparison of objective anxiety indicators

The VRD group suggested significantly higher heart rate during blood collection (*t* = 5.217, *p* < 0.001), longer crying duration (*t* = 6.384, *p* < 0.001), and greater cortisol change (*t* = 3.724, *p* < 0.001) compared to the CCT group. However, no between-group difference in heart rate was observed after blood collection (*t* = 1.839, *p* = 0.067). These findings suggest that the VRD group experienced elevated objective anxiety indicators. See Table 3 for details. The mean difference in crying duration (13.3 s) and the smaller rise in heart rate in the CCT group may be considered clinically meaningful in the context of a brief, stressful procedure.

**Table 3:** Comparison of objective anxiety indicators (Mean ± SD).

Indicator	CCT group (*n* = 110)	VRD group (*n* = 110)	Statistical value	*p*-value
Heart rate during blood collection (bpm)	112.6 ± 12.4	121.8 ± 13.7	*t* = 5.217	<0.001
Heart rate post-blood collection (bpm)	102.3 ± 11.2	104.9 ± 10.8	*t* = 1.839	0.067
Crying duration (seconds)	35.6 ± 12.8	48.9 ± 15.3	*t* = 6.384	<0.001
Cortisol change during blood collection (nmol/L)	3.2 ± 1.1	3.8 ± 1.3	*t* = 3.724	<0.001

### 3.4 Comparison of clinical procedural metrics

The CCT group suggested a significantly lower cooperation score (*U* = 2385.000, *p* < 0.001), a higher first-attempt venipuncture success rate (χ^2^ = 5.143, *p* = 0.023), and a shorter total blood collection time (*t* = 4.926, *p* < 0.001), indicating that CCT intervention markedly improved clinical procedural efficiency. Detailed data are presented in Table 4.

**Table 4:** Comparison of clinical procedural indicators.

Indicator	CCT group (*n* = 110)	VRD group (*n* = 110)	Statistical value	*p* value
Cooperation score	1.8 ± 0.7	2.4 ± 0.9	*U* = 385.000	<0.001
First-attempt success (*n*, %)	98 (89.1%)	91 (82.7%)	χ^2^ = 5.143	0.023
Total blood collection time (s)	126.8 ± 25.4	142.3 ± 28.7	*t* = 4.926	<0.001

### 3.5 Comparison of parental satisfaction

Parental satisfaction ratings were significantly higher in the CCT group (*U* = 2102.500, *p* < 0.001), suggesting greater acceptance of the CCT intervention among parents. The distribution of satisfaction levels is shown in Table 5.

**Table 5:** Comparison of parental satisfaction.

Satisfaction level	CCT group (*n* = 110)	VRD group (*n* = 110)	Statistical value	*p* Value
Very satisfied	45	32	*U* = 2102.500	<0.001
Satisfied	38	41		
Neutral	18	22		
Dissatisfied	6	11		
Very dissatisfied	3	4		

### 3.6 Logistic regression analysis of factors influencing anxiety reduction

Using anxiety reduction (defined as a ≥ 20% decrease in m-YPAS score during blood collection) as the dependent variable, multivariate logistic regression incorporating significant factors from univariate analysis (previous venipuncture experience, baseline anxiety, intervention type) identified CCT intervention as an independent factor associated with a higher likelihood of anxiety reduction (OR = 3.456, 95% CI: 1.892–6.312). Results are summarized in Table 6.

**Table 6:** Logistic regression analysis of factors associated with anxiety reduction.

Factor	B	SE	Wald	*p* Value	OR	95% CI
Intervention (CCT vs. VRD)	1.24	0.321	14.923	<0.001	3.456	1.892–6.312
Prior venipuncture experience	0.891	0.287	9.634	0.002	2.438	1.389–4.278
Baseline m-YPAS score	−0.056	0.018	9.876	0.002	0.945	0.912–0.979
Age	0.123	0.089	1.912	0.167	1.131	0.951–1.345

## 4 Discussion

Venipuncture-induced anxiety is highly prevalent among preschool-aged children (13). Non-pharmacological interventions, such as clown care therapy (CCT) and virtual reality distraction (VRD), have been widely implemented to alleviate medical procedure-related anxiety (14, 15); however, direct comparative studies remain limited. This retrospective cohort study aimed to compare the outcomes associated with Clown Care Therapy (CCT) and Virtual Reality Distraction (VRD) in mitigating procedure-related anxiety among preschool children undergoing venipuncture. While both non-pharmacological interventions suggest potential in pediatric anxiety management, direct head-to-head comparisons remain scarce, particularly within cultural contexts where humor perception and technology acceptance may introduce bias (16). Previous investigations have predominantly relied on subjective reports, lacking integration of objective physiological parameters and stratified analyses based on baseline anxiety levels, thereby limiting personalized application (17). To address these gaps, we implemented a multidimensional assessment framework combining validated subjective scales (m-YPAS) with objective biomarkers (heart rate, cortisol levels) in a tertiary hospital setting in China, thereby reducing measurement bias. The intervention leveraged the distinct mechanisms of CCT, which employs interactive play and music to foster positive engagement and immediate anxiolysis, and VRD, which primarily utilizes audiovisual diversion. CCT’s emphasis on social interaction may better align with the emotional needs of preschool children, potentiating its calming effects. Mechanistically, real-time interpersonal engagement in CCT is hypothesized to activate positive affective pathways and attenuate autonomic stress responses, whereas VRD’s efficacy, though notable, may be constrained by device dependency and cross-cultural adaptability. Rigorous standardized protocols ensured intervention fidelity, enhancing result reliability and generating high-quality evidence applicable to healthcare settings in China.

Regarding changes in anxiety scores, our findings suggested that the CCT group exhibited significantly lower anxiety levels during venipuncture compared to the VRD group. This difference may be attributed to the immediate interactive nature of CCT, where medical clowns’ humorous behaviors directly facilitate distraction and promote emotional regulation (18). In contrast, VRD relies on audiovisual immersion, whose effectiveness may be compromised by initial device-related discomfort or insufficient content suitability, particularly in younger children (19). Moreover, anxiety scores decreased rapidly post-venipuncture in both groups, indicating short-term anxiolytic effects for both interventions. The sustained advantage of CCT, however, suggests better adaptability to dynamic clinical scenarios, consistent with previous studies emphasizing the benefits of unstructured interactions (20). While some Western studies report favorable outcomes with VRD, our findings differed. We hypothesize that cultural and contextual factors, such as a possible greater familiarity with and preference for live human interaction over digital immersion in the studied population, might partially explain this discrepancy. However, this remains speculative and warrants direct investigation in cross-cultural studies (21).

Regarding objective anxiety indicators, the CCT group exhibited a lower heart rate increase, shorter crying duration, and reduced cortisol level fluctuations, confirming its superior efficacy in suppressing physiological stress responses (22). This divergence may stem from CCT’s multisensory engagement—incorporating musical and tactile interactions—which directly activates the parasympathetic nervous system to promote relaxation. In comparison, VRD’s unimodal sensory stimulation may be insufficient to counteract high-stress states in real time (23). The reduction in crying duration further reflects CCT’s behavioral regulatory advantages, likely associated with medical clowns’ immediate feedback and emotional support—a mechanism aligned with core non-pharmacological strategies in pediatric pain management (24). The cortisol findings suggest that CCT may more effectively buffer hypothalamic–pituitary–adrenal axis activation, minimize biomarker fluctuations, and thereby provide more stable anxiety control (25).

Comparative analysis of clinical procedural metrics revealed superior outcomes in the CCT group, characterized by enhanced child cooperation, higher first-attempt venipuncture success, and reduced total blood collection time, underscoring its practical utility in clinical workflows (26). Improved cooperation may stem from CCT’s interactive nature, which fosters trust and diminishes resistive behaviors, whereas VRD’s reliance on technology may occasionally disrupt nursing operations or child engagement (26). The increased first-attempt success rate is directly associated with reduced anxiety, as a calmer state facilitates easier venous identification and steadier needle insertion, thereby minimizing repeated attempts and attendant stress (27). Shortened procedure time not only enhances operational efficiency but may also reduce overall healthcare costs, aligning with initiatives to optimize care delivery in resource-constrained settings. These findings reinforce the value of CCT as a feasible intervention, particularly in high-volume pediatric contexts.

Parental satisfaction outcomes indicated higher ratings in the CCT group, reflecting stronger acceptance and approval of this non-technological intervention. This preference may arise from the humanistic elements of CCT, such as the clown performers’ approachability and entertainment value, which are readily perceived by parents as safe and engaging. In contrast, the technological nature of VRD may elicit concerns over screen exposure or device safety (28). The higher parental satisfaction with CCT could also be influenced by cultural or generational preferences for interpersonal engagement over technology-mediated interaction, although our study was not designed to test this specific hypothesis. Furthermore, the positive correlation between parental satisfaction and child anxiety reduction supports the importance of a family-centered care model, wherein parental emotional state influences child responsiveness (29).

Multivariate analysis identified CCT as an independent protective factor against anxiety, while baseline anxiety levels and previous venipuncture experience also significantly influenced outcomes (30). The advantage of CCT may be attributed to its personalized interaction, which adapts to a child’s immediate needs, whereas VRD’s standardized content lacks comparable flexibility (30). Children with higher baseline anxiety derived greater benefit from CCT, suggesting response heterogeneity and underscoring the need for stratified interventional strategies to optimize individualized application (31). Although prior venipuncture experience was positively correlated with reduced novelty-induced anxiety, CCT provided additional anxiolytic effects, highlighting its potential as a complementary intervention (32). These findings emphasize the necessity of incorporating patient-specific characteristics into intervention design, consistent with the principles of precision medicine (33).

### 4.1 Safety issues

Subjects did not report any side effects.

### 4.2 Study limitations

Our findings suggest the efficacy of CCT in reducing venipuncture-induced anxiety in preschool children, underscoring its clinical translatability. These results support the integration of CCT into standardized pediatric anxiety management guidelines for both outpatient and inpatient settings. Future directions include developing culturally adapted CCT protocols and training programs to improve accessibility. This study has several limitations. First, its retrospective, non-randomized design means that the association between CCT and better outcomes cannot be interpreted causally. Despite adjusting for multiple baseline covariates, unmeasured confounding (e.g., subtle differences in child temperament or parental anxiety that influenced the nurse’s choice of intervention) or confounding by indication remains a possibility. Second, the single-center design may limit generalizability. Third, the inherent variability in clown interactions, though guided by a protocol, and the inability to blind participants or staff are potential sources of bias. These limitations are counterbalanced by the study’s reflection of real-world clinical practice and its use of multiple objective outcome measures. Subsequent research should prioritize multicenter validation, long-term follow-up assessing anxiety recurrence, and economic evaluations to inform resource allocation.

## 5 Conclusion

In summary, this retrospective cohort analysis found that clown care therapy was associated with better outcomes than virtual reality distraction across multiple measures of venipuncture-related anxiety among preschool-aged children, as evidenced by improvements across subjective anxiety scores, objective physiological parameters, clinical procedural efficacy, and parental satisfaction. The interactivity and cultural congruence of CCT likely contribute to its advantages, whereas VRD, though effective, is limited by technological dependencies and individual variability. These findings support prioritizing CCT as a non-pharmacological anxiety management strategy in pediatric settings in China and warrant further research into intervention refinement and personalized application. Ultimately, this study provides an empirical foundation for enhancing pediatric medical experiences and advancing family-centered care.

## Data Availability

The datasets used in this study are available from the corresponding author upon reasonable request and with permission from the Ethics Committee of the First Affiliated Hospital of Anhui Medical University. Requests to access the datasets should be directed to JH, hongjf@ahmu.edu.cn.
